# Realizing the Embedded Growth of Large Li_2_O_2_ Aggregations by Matching Different Metal Oxides for High‐Capacity and High‐Rate Lithium Oxygen Batteries

**DOI:** 10.1002/advs.201700172

**Published:** 2017-07-20

**Authors:** Peng Zhang, Shoufeng Zhang, Mu He, Junwei Lang, Aimin Ren, Shan Xu, Xingbin Yan

**Affiliations:** ^1^ Laboratory of Clean Energy Chemistry and Materials State Key Laboratory of Solid Lubrication Lanzhou Institute of Chemical Physics Chinese Academy of Sciences Lanzhou 730000 P. R. China; ^2^ University of Chinese Academy of Sciences Beijing 100039 P. R. China; ^3^ State Key Laboratory of Theoretical and Computational Chemistry Jilin University Jilin 130023 P. R. China; ^4^ State Key Laboratory for Oxo Synthesis and Selective Oxidation Lanzhou Institute of Chemical Physics Chinese Academy of Sciences Lanzhou 730000 P. R. China

**Keywords:** discharge characteristics, Li_2_O_2_, LiO_2_ adsorption energy, Li‐O_2_ batteries, metal oxides

## Abstract

Large Li_2_O_2_ aggregations can produce high‐capacity of lithium oxygen (Li‐O_2_) batteries, but the larger ones usually lead to less‐efficient contact between Li_2_O_2_ and electrode materials. Herein, a hierarchical cathode architecture based on different discharge characteristics of α‐MnO_2_ and Co_3_O_4_ is constructed, which can enable the embedded growth of large Li_2_O_2_ aggregations to solve this problem. Through experimental observations and first‐principle calculations, it is found that α‐MnO_2_ nanorod tends to form uniform Li_2_O_2_ particles due to its preferential Li^+^ adsorption and similar LiO_2_ adsorption energies of different crystal faces, whereas Co_3_O_4_ nanosheet tends to simultaneously generate Li_2_O_2_ film and Li_2_O_2_ nanosheets due to its preferential O_2_ adsorption and different LiO_2_ adsorption energies of varied crystal faces. Thus, the composite cathode architecture in which Co_3_O_4_ nanosheets are grown on α‐MnO_2_ nanorods can exhibit extraordinary synergetic effects, i.e., α‐MnO_2_ nanorods provide the initial nucleation sites for Li_2_O_2_ deposition while Co_3_O_4_ nanosheets provide dissolved LiO_2_ to promote the subsequent growth of Li_2_O_2_. Consequently, the composite cathode achieves the embedded growth of large Li_2_O_2_ aggregations and thus exhibits significantly improved specific capacity, rate capability, and cyclic stability compared with the single metal oxide electrode.

## 1 Introduction

Rechargeable lithium‐oxygen (Li‐O_2_) batteries are triggering worldwide interest due to their ultrahigh theoretical energy density (3505 Wh kg^−1^ based on lithium peroxide (Li_2_O_2_)), thus exhibiting significant potential to meet the demand of long‐range electric vehicles.^1^, ^2^ In a typical Li‐O_2_ battery, during the discharging process, O_2_ reacts with Li^+^ to form Li_2_O_2_ with insulating and insoluble characteristics that lead to the gradual increase of electrode impedance until the electron transport cannot match the current density.^3^, ^4^, ^5^ For this reason, if conformal Li_2_O_2_ film or small Li_2_O_2_ aggregation is formed, the electrode surface will be passivated early and thus yield a low capacity.^6^, ^7^ If large‐sized Li_2_O_2_ aggregation is produced, the passivation of electrode surface will be prolonged and result in a relatively high capacity.^8^, ^9^, ^10^ Therefore, the capacity of Li‐O_2_ batteries is strongly related to the size of Li_2_O_2_ aggregation. For instance, Bruce and co‐workers and Luntz and co‐workers found that the size of Li_2_O_2_ toroids could be adjusted by changing the solvent or electrolyte additive, and the battery capacity could be increased with Li_2_O_2_ sizes.^6^, ^8^ Han and co‐workers reported that Li_2_O_2_ with large sheet‐like morphology provided a higher capacity than the small Li_2_O_2_ nanoparticles.^11^ Amine and co‐workers also found that large Li_2_O_2_ toroids delivered higher capacity than Li_2_O_2_ film.^12^ Thus, a large size is a desirable feature for Li_2_O_2_ in Li‐O_2_ batteries.

However, Chen and co‐workers and Byon and co‐workers both demonstrated that upsizing Li_2_O_2_ would result in a higher charging plateau and low charging rate.^13^, ^14^ That is, for large Li_2_O_2_ aggregations, a contradiction between high capacity and low oxygen evolution reaction (OER) overpotential is found. To solve this problem, many studies have focused on the development of OER catalysts. For example, Han co‐workers reported a PdCu/Super P cathode yielding large sheet‐like Li_2_O_2_ as well as low OER overpotential.^11^ Liu and co‐workers designed a carbon‐dotted/CoO/Super P cathode that could produce large Li_2_O_2_ toroids and perform lower overpotentials.^15^ Moreover, Peng and co‐workers have proved that the OER reaction interface of Li‐O_2_ batteries is electrode/Li_2_O_2_ instead of Li_2_O_2_/electrolyte.^4^ Basing on this result, further increasing contact sites between the catalyst and Li_2_O_2_, the decomposition of the large Li_2_O_2_ aggregations should become more easily and quickly. Meanwhile, large Li_2_O_2_ aggregations usually randomly deposit on the electrode surface,^16^, ^17^, ^18^, ^19^, ^20^ leading to the less effective contact between Li_2_O_2_ and electrode material. In this regard, confining the large Li_2_O_2_ aggregations in a hierarchical cathode/catalyst matrix should be an effective strategy to produce sufficient contact sites between them. Although many studies have focused on the construction of hierarchically structured cathodes,^21^, ^22^, ^23^, ^24^, ^25^ these reported structures still did not realize the strategy.

For the construction of the cathode architecture toward the embedded growth of Li_2_O_2_, using metal oxides is one of the ideal choices due to their scientific and practical values compared with noble metals, and their variety and better stability compared with carbon materials.^22^, ^26^, ^27^, ^28^, ^29^, ^30^ Among various metal oxides, MnO_2_ and Co_3_O_4_ are the most studied materials,^28^, ^31^, ^32^, ^33^, ^34^, ^35^, ^36^ and the corresponding results have shown that MnO_2_ and Co_3_O_4_ are promising active materials for constructing ideal cathode architecture.

Herein, we demonstrate that the embedded growth of large Li_2_O_2_ aggregations can be realized by constructing a hierarchical cathode architecture wherein Co_3_O_4_ nanosheets are grown on the surfaces of α‐MnO_2_ nanorods on a conductive carbon paper (CP). First, the different discharge characteristics of Co_3_O_4_ nanosheet and α‐MnO_2_ nanorod are analyzed through experimental observations and first‐principles calculations. α‐MnO_2_ nanorod tends to produce the uniform Li_2_O_2_ nucleation due to its preferential Li^+^ adsorption characteristic, resulting in the formation of uniform Li_2_O_2_ particles; whereas Co_3_O_4_ nanosheet tends to produce Li_2_O_2_ crystal seeds through the surface and solution due to its preferential oxygen adsorption characteristic, resulting in the separate formation of Li_2_O_2_ film and Li_2_O_2_ nanosheets. The composite cathode architecture wherein Co_3_O_4_ nanosheets are grown on α‐MnO_2_ nanorods exhibits extraordinary synergistic effects: α‐MnO_2_ can offer the initial Li_2_O_2_ nucleation sites and produce enough sites to grow ultrathin Co_3_O_4_ nanosheets, and Co_3_O_4_ nanosheets are conducive to yield dissolved LiO_2_. Thus, large mooncake‐like Li_2_O_2_ and sheet‐like Li_2_O_2_ both possessing embedded structures are formed on CP‐MnO_2_‐Co_3_O_4_ electrode at low and high current densities, respectively. As a consequence, such composite cathode exhibits remarkably improved electrochemical performance compared with the single ones, in terms of a large capacity (5950 mAh g^−1^ at 51 mA g^−1^), outstanding rate capability (2574 mAh g^−1^ at 1.03 A g^−1^), as well as good cyclic stability (54 cycles at the limited capacity of 1000 mAh g^−1^).

## 2 Results and Discussion

### 2.1 Structure Characterization of CP‐MnO_2_ and Its Discharge Products in Li‐O_2_ Cells

**Figure 1:**
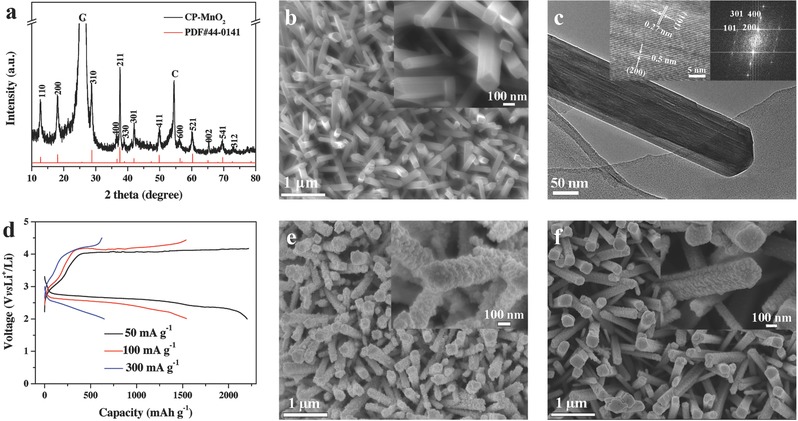
a) XRD pattern and b) SEM image of CP‐MnO_2_. c) TEM image of an individual MnO_2_ nanorod. Insets are the HRTEM image and its corresponding Fourier transform image. d) Charge–discharge curves of CP‐MnO_2_ at different currents. SEM images of CP‐MnO_2_ electrode discharged at e) 104 mA g^−1^ and f) 311 mA g^−1^. The insets in b), e), and f) are the corresponding magnified images.

In this study, α‐MnO_2_ and Co_3_O_4_ were directly grown on CP substrates as freestanding electrodes to avoid using any binder that will increase the resistance and induce the possible side reactions in Li‐O_2_ cells.^37^, ^38^ Specifically, CP‐MnO_2_ was prepared via a facile hydrothermal method and its microstructure and morphology are shown in **Figure** **1**. X‐ray diffraction (XRD) pattern presented in Figure 1a reflects that the diffraction peaks can almost be well indexed to tetragonal α‐MnO_2_ (PDF#44‐0141) except the carbon peaks (26° and 53°). As shown in Figure 1b, scanning electron microscopy (SEM) images exhibit a uniform growth of highly ordered nanorods array on the CP substrate. The transmission electron microscopy (TEM) image further confirms the nanorod‐like morphology (Figure 1c) and the selected area electron diffraction (SEAD, Figure S1a, Supporting Information) suggests the single‐crystal structure of α‐MnO_2_ nanorod. Lattice fringes displayed in the high‐resolution TEM (HRTEM) are ≈0.5 and 0.27 nm, corresponding to the (200) and (101) planes, respectively. Combined with the Fourier transform image (inset of Figure 1c), the growth direction of α‐MnO_2_ nanorod can be confirmed to be at the [001] axis with the exposed planes of (020) and (110). When employed as the cathode for Li‐O_2_ battery, the CP‐MnO_2_ electrode delivers specific capacities of 2195, 1543, and 648 mAh g^−1^ at 52, 104, and 311 mA g^−1^, respectively. Correspondingly, the ORR (OER) voltage plateaus are ≈2.63 V (4.05 V), 2.52 V (4.15 V), and 2.40 V (4.11 V) at different currents (Figure 1d).

As mentioned before, the morphology of Li_2_O_2_ can reflect the electrochemical performance of a Li‐O_2_ battery. Here, the discharge products were examined by SEM and XRD. Figure 1e,f shows CP‐MnO_2_ electrode morphology discharged at 104 and 311 mA g^−1^, respectively. The surfaces of MnO_2_ nanorods in two samples are both covered by numerous particles except for the different thicknesses. Here a thicker layer represents a higher capacity value. The XRD pattern of the discharged CP‐MnO_2_ exhibits the characteristic peaks of Li_2_O_2_ at about 32.7° and 35° and they disappear after recharging (Figure S2a, Supporting Information), indicating the reversibility of CP‐MnO_2_ electrode. The weak diffraction peaks of Li_2_O_2_ may be attributed to its low crystallinity and low content in this sample, which is also reflected by other metal oxide electrodes.^39^, ^40^, ^41^ Furthermore, the obvious peak shift of MnO_2_ to the low angle direction can be detected from the discharged electrode, suggesting the volume increase of the lattice.^41^, ^42^ The increased lattice volume should be attributed to the storage of Li*_x_*O*_y_* in 2 × 2 MnO_6_ octahedron tunnels of α‐MnO_2_ instead of Li^+^ insertion, which is due to the insertion of Li*_x_*O*_y_* clogging the channels and Li_2_O_2_ on the surfaces of MnO_2_ further covering the channels.^31^, ^42^, ^43^ After recharging, these peaks can shift back to their original positions, suggesting the reversible extraction of the Li*_x_*O*_y_* in the tunnels. Additionally, the electrochemical performance of the pure CP substrate was also investigated to confirm the major contribution of the loading active materials on battery capacity (Figure S3, Supporting Information).

### 2.2 Structure Characterization of CP‐Co_3_O_4_ and Its Discharge Products in Li‐O_2_ Cells

**Figure 2:**
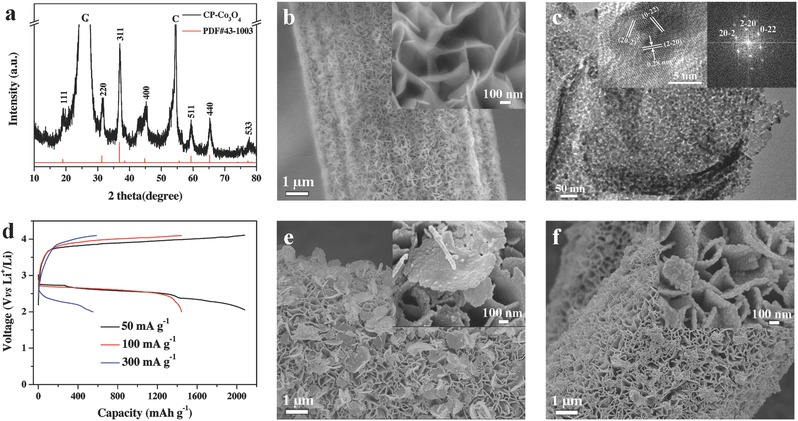
a) XRD pattern and b) SEM image of CP‐Co_3_O_4_. c) TEM image of an individual Co_3_O_4_ nanosheet. The insets are the HRTEM image and its corresponding Fourier transform image. d) Charge–discharge curves of CP‐Co_3_O_4_ electrode at different currents. SEM images of CP‐Co_3_O_4_ electrode discharged at e) 102 mA g^−1^ and f) 306 mA g^−1^. The insets in b), e), and f) are the corresponding magnified images.

CP‐Co_3_O_4_ electrode was synthesized via a simple electro‐deposition method followed by an annealing process (Figure S4, Supporting Information). Its structure characteristics and electrochemical performance were analyzed and the corresponding results are presented in **Figure** **2**. As shown in Figure 2a, the major XRD peaks of CP‐Co_3_O_4_ sample are consistent with the pure Co_3_O_4_ phase (PDF#43‐1003) except for the diffraction peaks of CP. SEM images show the uniform and vertical growth of Co_3_O_4_ nanosheets on the CP skeleton, and these nanosheets interconnect into a 3D framework (Figure 2b). TEM images verify that such a Co_3_O_4_ nanosheet has an obvious surface‐porous structure (Figure 2c and Figure S5a, Supporting Information) and the nitrogen adsorption–desorption isotherms prove the large specific surface area of Cp‐Co_3_O_4_ sample (74.73 m^2^ g^−1^, Figure S6b, Supporting Information). The diffraction rings in the SEAD pattern (Figure S1b, Supporting Information) of the Co_3_O_4_ nanosheet indicate its polycrystalline structure, and the HRTEM and Fourier transform images (insets of Figure 2c) further show that the polycrystalline nanosheet is composed of numerous Co_3_O_4_ monocrystals. When employed as the cathode for a Li‐O_2_ battery, the CP‐Co_3_O_4_ electrode exhibits specific capacities of 2080, 1445, and 551 mAh g^−1^ at 51, 102, and 306 mA g^−1^, respectively (Figure 2d). These profiles also reveal that the discharge (charge) plateau voltages are 2.59 V (3.92 V), 2.60 V (3.98 V), and 2.27 V (3.95 V). The electrochemical properties shown here are similar to previously reported freestanding Co_3_O_4_ electrodes.^44^, ^45^, ^46^ XRD results of discharged and charged CP‐Co_3_O_4_ electrodes prove the reversible formation and decomposition of Li_2_O_2_ (Figure S2b, Supporting Information). Moreover, as displayed in Figure 2e, after the electrode discharged at 100 mA g^−1^, numerous Li_2_O_2_ nanosheets are generated on the electrode surfaces with a randomly floating state. Simultaneously, film‐like Li_2_O_2_ is also formed on the surfaces of Co_3_O_4_ nanosheets (seen from the inset). In comparison, when the current increases to 300 mA g^−1^, such nanosheet‐like morphology disappears from the electrode. Instead, film‐like Li_2_O_2_ and discrete nanoparticle aggregations occupy the electrode surface.

### 2.3 Discharge Characteristics of CP‐MnO_2_ and CP‐Co_3_O_4_

The morphology of the discharge product depends on the electrode material morphology, current density, and the intrinsic discharge characteristic of used electrode material. For the influence of electrode morphology, previous studies have reported that carbon nanotubes and granular KB carbon can both produce toroidal Li_2_O_2_. The morphology of electrode material is not the critical factor to determine its discharge characteristic.^9^, ^47^ Our recent study also reported that flower‐like Li_2_O_2_ assembled by Li_2_O_2_ sheets was both produced on Ni/Co_3_O_4_ nanowires and Ni/Co_3_O_4_ rectangular nanosheet electrodes.^48^ In this study, aside from setting the same mass current density for testing CP‐MnO_2_ and CP‐Co_3_O_4_ electrodes, we also compared the discharged morphology of the two electrodes at the same current density of ≈1.40 mA m^−2^_SSA_ based on the specific surface area (Table S1 and Figure S7, Supporting Information). As a result, the morphology of the discharged CP‐MnO_2_ electrode still exhibits uniform granular Li_2_O_2_ coating on α‐MnO_2_ nanorod surface. Thus, the intrinsic characteristic of metal oxides determines the growth process of Li_2_O_2_ in an operated Li‐O_2_ cell. Then, we deeply analyzed the different discharge characteristics of α‐MnO_2_ nanorod and Co_3_O_4_ nanosheet, respectively.

**Figure 3:**
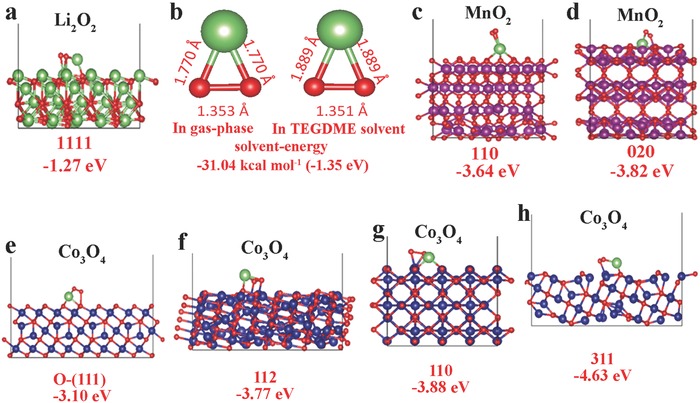
a) The adsorption energy of LiO_2_ on (1111) plane of Li_2_O_2_. b) The solvation energy of LiO_2_ in Tetraethylene glycol dimethyl ether (TEGDME). c,d) The adsorption energies of LiO_2_ on (110) and (020) planes of α‐MnO_2_. e–h) The adsorption energies of LiO_2_ on O‐(111), (112), (110), and (311) planes of Co_3_O_4_.

Schematic illustrations of the discharging process of α‐MnO_2_ (Figure S8a, Supporting Information) and Co_3_O_4_ (Figure S8b, Supporting Information) and the corresponding equations were outlined. For α‐MnO_2_, the first ORR step was proven to be a Li^+^ adsorption process with desolvation (Equation (S1) in Figure S8 of the Supporting information),^31^, ^32^, ^49^ obtaining an electron and further adsorbing O_2_ to form LiO_2_* (“*” represents adsorbed species) at the MnO_2_ surface (Equation (S2) in Figure S8 of the Supporting information).^50^, ^51^ Then, LiO_2_* transforms to Li_2_O_2_ through an electrochemical reduction or a disproportionation reaction (Equation (S3) in Figure S8 of the Supporting information).^50^, ^52^ Owing to the single crystal structure of α‐MnO_2_ nanorods, the accommodation of Li_2_O_2_ in 2 × 2 MnO_6_ octahedron tunnels, and close LiO_2_ adsorption energy for exposed crystal face of α‐MnO_2_ nanorod (**Figure** **3**c,d),^31^, ^42^ the formed Li_2_O_2_ seeds can be uniformly distributed on the MnO_2_ surface and act as the desired seeds for subsequent Li_2_O_2_ growth. After the nucleation process of Li_2_O_2_ on α‐MnO_2_ surface, LiO_2_ can be adsorbed on the Li_2_O_2_ surface or dissolved into electrolyte due to the close adsorption energy of LiO_2_ on Li_2_O_2_ (−1.27 eV, Figure 3a) and the solvation energy of LiO_2_ (−1.35 eV, Figure 3b) (Equation (S4) in Figure S8 of the Supporting information). Thus, the uniform Li_2_O_2_ nucleus can induce the subsequent growth of Li_2_O_2_ simultaneously through surface and solution, finally resulting in the formation of Li_2_O_2_ particles (Equations (S5) and (S6) in Figure S8 of the Supporting information).

For CP‐Co_3_O_4_ electrode, the first ORR step is oxygen adsorption followed by oxygen reduction to form O_2_^‐^ on the electrode surface, and then O_2_^‐^ transfers to electrolyte due to solvation (Equations (S7) and (S8) in Figure S8 of the Supporting information).^10^, ^33^, ^53^ The dissolved O_2_^‐^_sol_ (“sol” represents solvated species) and Li^+^ form LiO_2sol_ by coupling (Equation (S9) in Figure S8 of the Supporting information).^54^ Near the electrode surface, the coupled LiO_2sol_ can be adsorbed on Co_3_O_4_ nanosheet surfaces owing to the larger adsorption energy between them (−3.10 to −4.63 eV on different crystal planes, Figure 3e–h) compared to the solvation energy of LiO_2_ in electrolyte (−1.35 eV, Equation (S10) in Figure S8 of the Supporting information). Then, the adsorbed LiO_2_* undergoes a second reduction or disproportionation to form Li_2_O_2_ as sur‐seeds (“sur‐seeds” represent the seeds formed through surface, Equation (S11) in Figure S8 of the Supporting information).^55^ Simultaneously, in the bulk electrolyte, LiO_2sol_ transforms to Li_2_O_2_ through disproportionation (Equation (S12) in Figure S8 of the Supporting information).^12^ When Li_2_O_2_ cluster reaches a supersaturated state, solid Li_2_O_2_ deposits on the electrode surface as sol‐seeds (“sol‐seeds” represent the seeds formed through solution).^6^, ^19^ Thus, for Co_3_O_4_ material, the nucleation occurs simultaneously through surface and solution, which leads to further growth of Li_2_O_2_ on the surface and in the electrolyte.

### 2.4 Design Philosophy for the Composite Cathode

**Figure 4:**
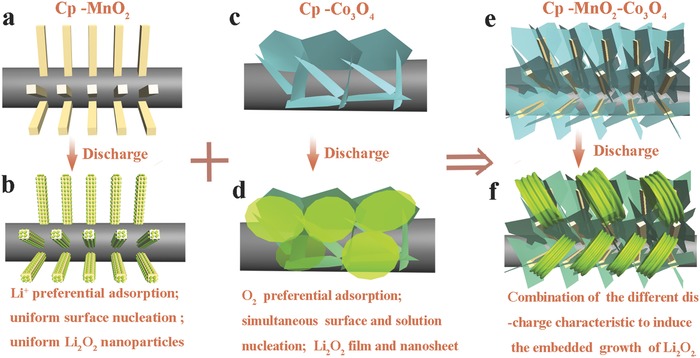
e) Schematic illustration of designing the composite cathode architecture f) with ideal discharge product morphology based on different discharge characteristics of a,b) CP‐MnO_2_ and c,d) CP‐Co_3_O_4_.

Basing on the above analyses, the nucleation processes of Li_2_O_2_ for α‐MnO_2_ and Co_3_O_4_ are quite different. α‐MnO_2_ undergoes Li^+^ adsorption and O_2_ reduction to directly form LiO_2_ on MnO_2_ surfaces. Co_3_O_4_ undergoes O_2_ adsorption, O_2_ reduction, O_2_^‐^ solvation, coupling of O_2_^‐^ and Li^+^, and then forms dissolved LiO_2_ oligomer or being adsorbed on electrode surfaces. Thus, the nucleation rate of α‐MnO_2_ should be faster than Co_3_O_4_ at the initial discharge process. Meanwhile, for Co_3_O_4_ electrode, the growth of Li_2_O_2_ through surface and electrolyte occurs at the same time. Taking these findings into account, the composite electrode, i.e., CP‐MnO_2_‐Co_3_O_4_ (**Figure** **4**e), may combine the discharge characteristics of α‐MnO_2_ and Co_3_O_4_ (Figure 4a–d), of which α‐MnO_2_ nanorods function as the initial growth sites of Li_2_O_2_ acting as the seeds for subsequent Li_2_O_2_ growth and simultaneously provide plenty of sites for Co_3_O_4_ nanosheets deposition inducing the formation of sufficient activity area to provide a large amount of dissolved LiO_2_. Then, as shown in Figure 4f, Li_2_O_2_ produced on the composite cathode architecture probably possesses a large size and an embedded structure, resulting in the remarkable improvement of battery performance.

### 2.5 Structure Characterization of CP‐MnO_2_‐Co_3_O_4_

**Figure 5:**
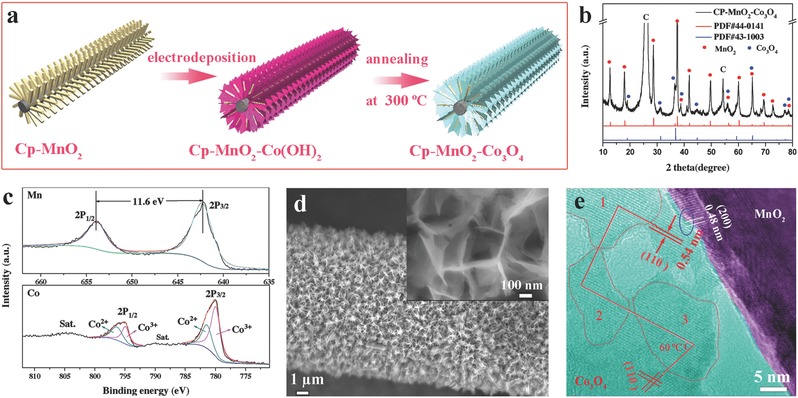
a) Schematic of the preparation of CP‐MnO_2_‐Co_3_O_4_. b) XRD pattern of CP‐MnO_2_‐Co_3_O_4_. c) XPS Mn 2p and Co 2p spectra of CP‐MnO_2_‐Co_3_O_4_. d) SEM image of a single fiber in the composite electrode. The inset shows the layout of Co_3_O_4_ nanosheets on a MnO_2_ nanorod from the top view. e) TEM image of the interface between MnO_2_ nanorod and Co_3_O_4_ nanosheet.

As shown in **Figure** **5**a, the CP‐MnO_2_‐Co_3_O_4_ composite electrode was synthesized through electro‐depositing Co_3_O_4_ on the CP‐MnO_2_ substrate followed by an annealing process. Figure 5b displays the XRD pattern of as‐prepared composite electrode, presenting the diffraction peaks of both MnO_2_ and Co_3_O_4_. X‐ray photoelectron spectroscopy (XPS) survey also confirms the coexistence of MnO_2_ and Co_3_O_4_ (Figure 5c and Figure S9, Supporting Information). For Mn 2p spectrum, the Mn 2p_3/2_ (642.1 eV) and Mn 2p_1/2_ (653.8 eV) peaks have a spin‐energy separation of 11.7 eV, reflecting the +4 oxidation state for Mn.^56^ For Co 2p spectrum, peaks at 780.5 and 796.6 eV correspond to Co 2p_3/2_ and Co 2p_1/2_, respectively, indicating the characteristic of Co_3_O_4_ phase.^57^ Fitting using Gaussian method, the Co 2p spectrum can be well fitted into two spin orbits with features of Co^2+^ and Co^3+^. From the top view of SEM images, Co_3_O_4_ nanosheets interconnect each other to form numerous matrices for the accommodation of Li_2_O_2_ during the discharge process (Figure 5d and inset). From the side view (Figure S10, Supporting Information), Co_3_O_4_ nanosheets grow on the surface of each MnO_2_ nanorod from bottom to top, but these nanosheets do not cover the whole surface of MnO_2_ nanorods. The microstructure of Co_3_O_4_ nanosheets grown on a MnO_2_ nanorod was further revealed by TEM (Figure 5e and Figure S11, Supporting Information). As shown in a typical interface area marked with a blue circle in Figure 5e, the main lattice fringes spacing are 0.48 and 0.54 nm, corresponding to the (002) plane of MnO_2_ and the (110) plane of Co_3_O_4_, respectively. Moreover, as seen from the crystal planes of the Co_3_O_4_ nanosheet (their boundaries are marked with a dashed line), their crystal orientations are the same (marked with a red solid line). Nevertheless, the Co_3_O_4_ nanosheets in composited electrode still exhibit polycrystalline structure (Figure S12, Supporting Information).

Furthermore, mesopores on Co_3_O_4_ nanosheet in the composite electrode are larger than those in CP‐Co_3_O_4_ electrode (Figure S5b, Supporting Information). The formation of these larger mesopores is due to much thinner Co_3_O_4_ nanosheets in CP‐MnO_2_‐Co_3_O_4_ sample compared with the ones in CP‐Co_3_O_4_ under the same annealing process (Figure S13, Supporting Information).^58^ With the consideration of the identical mass of Co_3_O_4_ on the two electrodes, the decreased thickness of Co_3_O_4_ nanosheets is linked to the significantly increased height derived from more depositing sites on the CP‐MnO_2_ substrate than those on the pure CP substrate (Figure S14, Supporting Information). As a result, the composite electrode exhibited a large surface area of 71.18 m^2^ g^−1^ (Figure S6c, Supporting Information), which would be in favor of producing a large amount of dissolved LiO_2sol_. Furthermore, the abundant mesoporous structure of the Co_3_O_4_ nanosheets may also facilitate the transport of electrolyte, oxygen, superoxide, and peroxide species. The surface area of the composite electrode is slightly lower than that of CP‐Co_3_O_4_ electrode (74.73 m^2^ g^−1^), mainly because of the low specific surface area (21.31 m^2^ g^−1^), high mass loading of MnO_2_ (0.85 mg cm^−2^ for MnO_2_; 0.51 mg cm^−2^ for Co_3_O_4_), larger mesoporous size of Co_3_O_4_, and partially overlapped surface of MnO_2_ and Co_3_O_4_ in the composite electrode.

### 2.6 Performance Comparison of CP‐MnO_2_, CP‐Co_3_O_4_ and CP‐MnO_2_‐Co_3_O_4_ Cathodes, and the Discharge Products of CP‐MnO_2_‐Co_3_O_4_ Electrode

**Figure 6:**
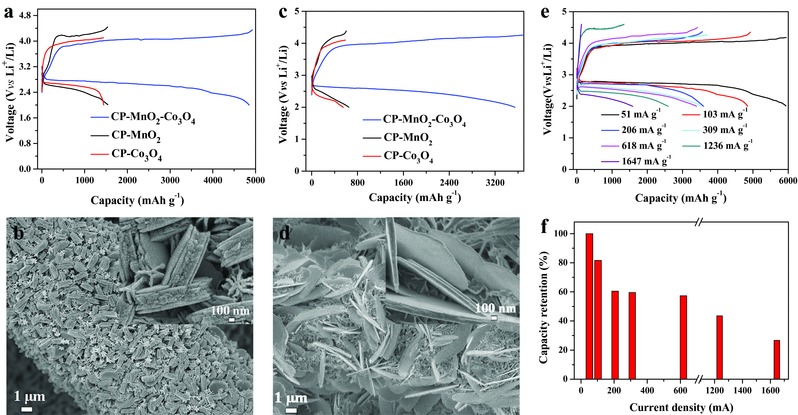
Charge–discharge curves of three electrodes at a) ≈103 mA g^−1^ and c) ≈309 mA g^−1^, respectively. SEM images of discharged CP‐MnO_2_‐Co_3_O_4_ electrode at b) 103 mA g^−1^ and d) 309 mA g^−1^, respectively. e) Rate capability of the Li‐O_2_ battery with CP‐MnO_2_‐Co_3_O_4_ electrode at different current densities. f) The capacity retention of CP‐MnO_2_‐Co_3_O_4_ electrode at different current densities.

The electrochemical performance of Li‐O_2_ battery with CP‐MnO_2_‐Co_3_O_4_ electrode was then investigated and the results are shown in **Figure** **6**. By comparing the charge–discharge curves of the three electrodes, it is clearly seen that CP‐MnO_2_‐Co_3_O_4_ electrode exhibits a much higher capacity (4850 mAh g^−1^) than the two other electrodes (CP‐MnO_2_ and CP‐Co_3_O_4_) at ≈103 mA g^−1^. SEM image of the discharged CP‐MnO_2_‐Co_3_O_4_ electrode was investigated. As expected, mooncake‐like Li_2_O_2_ is uniformly embedded in the given matrices (Figure 6b) and the formation of a large amount of Li_2_O_2_ aggregation endows the higher capacity of CP‐MnO_2_‐Co_3_O_4_ electrode. Such capacity improvement is more obvious at a high current density. As shown in Figure 6c, the composite electrode can deliver 3543 mAh g^−1^ at 309 mA g^−1^, almost six times higher than the two other electrodes. After discharging, numerous large Li_2_O_2_ sheets form on the surface of CP‐MnO_2_‐Co_3_O_4_ electrode with an inlaid structure (Figure 6d). Moreover, as shown in Figure 6e,f, the CP‐MnO_2_‐Co_3_O_4_ electrode exhibits superior rate capability. For instance, the discharge capacity is 3401 mAh g^−1^ at 618 mA g^−1^, 57.3% of the capacity at 51 mA g^−1^ (5940 mAh g^−1^). When the current increases to 1236 mA g^−1^, the capacity still reaches 2592 mAh g^−1^, 43.6% of the initial capacity. Additionally, we also compared the electrochemical performance of the three samples normalized by their electrode area, and the related results are shown in Table S2 and Figure S15 (Supporting Information). The results show that the performance improvement of the composite electrode is more remarkable when the test results are normalized by the electrode area, further reflecting the effectivity of this cathode architecture.

We believe that the remarkably improved electrochemical performance should be attributed to the synergistic effect of the different discharge characteristics of MnO_2_ and Co_3_O_4_ through the scientific design of the cathode architecture. In detail, the obviously increased height of Co_3_O_4_ nanosheets in the vertical direction can significantly increase the amount of LiO_2sol_ and boost the growth of Li_2_O_2_, resulting in the formation of large aggregations (mooncake‐like and large sheet‐like Li_2_O_2_). Simultaneously, these Li_2_O_2_ aggregations are embedded in the cathode architecture, which can increase the contact sites between electrode materials and Li_2_O_2_ with a result of reduced overpotential and significantly improved rate capability of Li‐O_2_ cell. Furthermore, during the discharge process, Li^+^ can intercalate into MnO_2_ crystal structure, as proven from the weaker peak shift in XRD pattern of discharged CP‐MnO_2_‐Co_3_O_4_ electrode than that in XRD pattern of discharged CP‐MnO_2_ electrode (Figures S16 and S2, Supporting Information). The decreased peak shift to the low angle indicates the reduced volume change during the discharge process, suggesting the reduced amount or shrinking volume of insert species. As seen from Figure S17 (Supporting Information), the close capacity values between CP‐MnO_2_ and CP‐MnO_2_‐Co_3_O_4_ tested in argon atmosphere indicate that Li^+^ can diffuse across the Co_3_O_4_ nanosheets (the specific capacity values of two electrodes were both calculated based on the mass of MnO_2_). Simultaneously, the O_2_ adsorption characteristic of Co_3_O_4_ can lead to the low availability of O_2_ on MnO_2_ surfaces. Thus, in this case, the inserted Li*_x_*O*_y_* is partially replaced by Li^+^ due to the overlapped interface that can allow Li^+^ diffusion but prevent O_2_ transmission. Fortunately, the lithiated MnO_2_ was proved to be a better electronic conductor than the pristine MnO_2_, facilitating the charge transfer during electrochemical reactions.^41^ Additionally, to confirm the key role of MnO_2_ in the composite structure, another composite electrode with a higher Co_3_O_4_ loading was prepared and its SEM images are presented in Figure S18a,b (Supporting Information). When the deposition quantity of Co_3_O_4_ increases from 1 to 2.5 C cm^−2^, the surface of MnO_2_ sample is completely covered by Co_3_O_4_ nanosheets. After discharging, this electrode can also form abundant Li_2_O_2_, resulting in a high discharge capacity (Figure S18c, Supporting Information). However, the discharge products do not embed in the matrices of the electrode, leading to a larger OER overpotential compared with the composite electrode with an appropriate Co_3_O_4_ loading (Figure S18c,d, Supporting Information).

**Figure 7:**
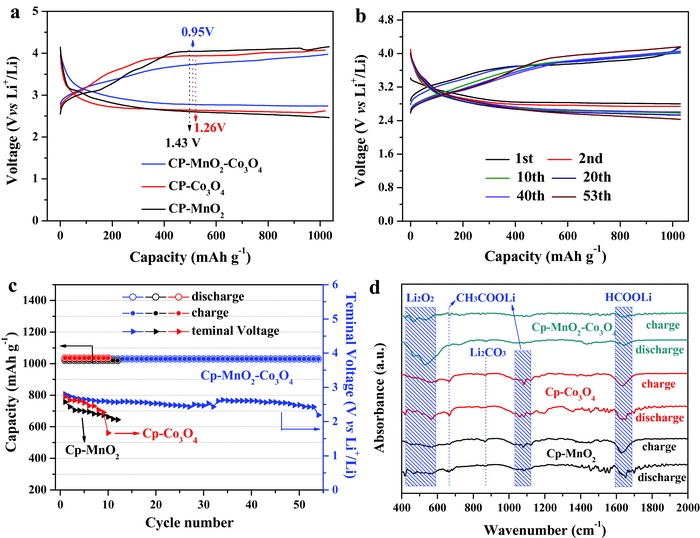
a) Charge–discharge profiles of CP‐MnO_2_, CP‐Co_3_O_4_, and CP‐MnO_2_‐Co_3_O_4_ electrodes at ≈103 mA g^−1^ with a limited capacity of ≈1030 mAh g^−1^. b) Charge–discharge profiles of CP‐MnO_2_‐Co_3_O_4_ electrode at different cycles during cyclic tests. c) Cycling performance of the three electrodes measured at ≈103 mA g^−1^ under the limited capacity of ≈1030 mAh g^−1^. d) FTIR spectra of the three electrodes at the eighth cycle.

As shown in **Figure** **7**a, CP‐MnO_2_‐Co_3_O_4_ electrode exhibits the lowest overpotential among the three electrodes during the limited capacity test. Meanwhile, no obvious change exists in the cyclic charge–discharge profile (Figure 7b), reflecting its good cycle stability. In comparison, CP‐MnO_2_‐Co_3_O_4_ electrode is able to perform over 50 cycles before the terminal voltage decreases below 2 V, while CP‐MnO_2_ and CP‐Co_3_O_4_ electrodes can only achieve approximately ten cycles at a limited capacity of ≈1030 mAh g^−1^ (Figure 7c). Fourier Transform in frared (FTIR) spectra of the three electrodes at the eighth charge–discharge cycle were measured to investigate the reversibility of the three electrodes. As shown in Figure 7d, the obvious existence of the peaks around 400 to 600 cm^−1^ indicates the reversible formation and decomposition of Li_2_O_2_ for the CP‐MnO_2_‐Co_3_O_4_ composite electrode. For CP‐MnO_2_‐Co_3_O_4_ electrode, the weakest intensity of organic lithium salt peaks suggests the slightest electrolyte decomposition. Also, the peak of Li_2_CO_3_ at 870 cm^−1^ is observed for CP‐MnO_2_ and CP‐Co_3_O_4_ electrodes. The formation of Li_2_CO_3_ is mainly due to the oxidation of carbon during the charge process.^59^ Thus, this peak may derive from the side reaction related to CP substrate. As seen from FTIR spectra of CP‐MnO_2_‐Co_3_O_4_ electrode at the 30th cycle (Figure S19, Supporting Information), the reversible decomposition of Li_2_O_2_ can also be detected. However, the intensity of peaks for organic lithium salt increases at the same time, indicating the gradual accumulation of the byproducts on the composite cathode, which further leads to the passivated electrode surfaces and the limited cycle life (54 cycles for CP‐MnO_2_‐Co_3_O_4_ electrode).

Because the discharge depth can also affect the cycling performance of Li‐O_2_ batteries, we further compared the cyclic stability of the three electrodes under the identical rate of limited discharge capacity to their full capacities. In detail, the capacity of CP‐MnO_2_‐Co_3_O_4_ electrode was 4850 mAh g^−1^ at 103 mA g^−1^ and 1030 mAh g^−1^ (≈21% of the full capacity) was chosen as the limited capacity during the cyclic tests. Here, this discharge ratio was also applied in CP‐MnO_2_ and CP‐Co_3_O_4_ electrodes (1543 mAh g^−1^ × 21% = 323 mAh g^−1^, with the value of 320 mAh g^−1^ chosen for the two electrodes). As shown in Figure S20 (Supporting Information), the cyclic stability of CP‐MnO_2_ and CP‐Co_3_O_4_ electrodes can be remarkably improved compared with that of the electrodes under the limited capacity setting at ≈1030 mAh g^−1^. Even so, their cycle lives are still inferior to CP‐MnO_2_‐Co_3_O_4_ electrode. Moreover, we compared the morphologies of the three electrodes after recharging (Figure S21, Supporting Information). α‐MnO_2_ nanorods are entirely covered by byproducts after being charging at the 25th cycle (may have some undecomposed Li_2_O_2_, Figure S21a, Supporting Information) and Co_3_O_4_ surfaces are partially covered by byproducts (Figure S21b, Supporting Information). Fortunately, no obvious byproduct was found on the charged CP‐MnO_2_‐Co_3_O_4_ electrode at 25th cycle (Figure S21c, Supporting Information). However, after recharging at the 54th cycle, undesired byproducts also covered on CP‐MnO_2_‐Co_3_O_4_ electrode surfaces (Figure S21d, Supporting Information). These results show that the composite electrode is conducive to alleviate the accumulation of byproducts, thus exhibiting enhanced cyclic stability. Consequently, when the composite electrode was tested at the limited capacity of 320 mAh g^−1^, it can achieve 142 cycles before the discharged terminal voltage decreases below 2 V (Figure S22, Supporting Information).

## 3 Conclusions

In summary, a cathode architecture composed of nanostructural α‐MnO_2_ and Co_3_O_4_ was developed to achieve embedded growth of discharge product Li_2_O_2_. To this end, the inherent catalytic characteristics of α‐MnO_2_ and Co_3_O_4_ are first studied through experimental observations and first‐principle calculations. For the single MnO_2_ and Co_3_O_4_, the CP‐MnO_2_ electrode produces Li_2_O_2_ with granular morphology due to its preferential Li^+^ adsorption property, accommodating Li*_x_*O*_y_* in 2 × 2 MnO_6_ octahedron channels and similar LiO_2_ adsorption energy of exposed crystal faces. The CP‐Co_3_O_4_ electrode produces nanosheet‐like Li_2_O_2_ as well as film‐like Li_2_O_2_ on the electrode surface owing to its preferential O_2_ adsorption characteristic and the difference in LiO_2_ adsorption energy of exposed crystal faces. On this basis, a hierarchical cathode architecture where Co_3_O_4_ nanosheets attach on MnO_2_ nanorods array was fabricated, and the as‐made composite electrode exhibits a distinct synergistic effect between α‐MnO_2_ and Co_3_O_4_. On the one hand, α‐MnO_2_ acts as the initial growing sites. On the other hand, ultrathin Co_3_O_4_ nanosheets with obviously increased height further produce a large amount of dissolved LiO_2_. Thus, uniform mooncake‐like Li_2_O_2_ and large sheet‐like Li_2_O_2_ can be generated on the composite electrode with an embedded structure at low and high current densities, respectively. As a consequence, this composite electrode can offer remarkably improved electrochemical performance when compared to the single α‐MnO_2_ or Co_3_O_4_ electrode, in terms of high reversible capacity, superior rate capability, and outstanding cycle stability. This study develops a feasible strategy to design reasonable cathode architectures for realizing the embedded growth of Li_2_O_2_. It also provides valuable information for understanding the discharge mechanism of Li‐O_2_ batteries and designing high‐performance Li‐O_2_ cathodes. Notably, this design philosophy may also be interesting for other energy storage devices.

## Conflict of Interest

The authors declare no conflict of interest.

## Supporting information

Supplementary
