# Supplementary material for: Realizing the Embedded Growth of Large Li2O2 Aggregations by Matching Different Metal Oxides for High‐Capacity and High‐Rate Lithium Oxygen Batteries
Source: Adv Sci (Weinh). 2017 Jul 20;4(11):1700172. doi: 10.1002/advs.201700172 (PMC5700630; doi:10.1002/advs.201700172)
Supplement: Supplementary file 1 — Supplementary [file ADVS-4-na-s001.pdf]

Copyright WILEY-VCH Verlag GmbH & Co. KGaA, 69469 Weinheim, Germany, 2017.

ADVANCED SCIENCE
Open Access

Supporting Information

for *Adv. Sci.,* DOI: 10.1002/advs.201700172

Realizing the Embedded Growth of Large Li_2O_2 Aggregations by Matching Different Metal Oxides for High-Capacity and High-Rate Lithium Oxygen Batteries

Peng Zhang, Shoufeng Zhang, Mu He, Junwei Lang, Aimin Ren, Shan Xu, and Xingbin Yan**

Copyright WILEY-VCH Verlag GmbH & Co. KGaA, 69469 Weinheim, Germany, 2013.

Supporting Information

Realizing the Embedded Growth of Large Li_2O_2 Aggregations by Matching Different Metal Oxides for High-Capacity and High-Rate Lithium Oxygen Batteries

Peng Zhang, Shoufeng Zhang, Mu He, Junwei Lang, Aimin Ren, Shan Xu, and Xingbin Yan**

Dr. P. Zhang, Dr. J. W. Lang, Prof. X.B. Yan

Laboratory of Clean Energy Chemistry and Materials, State Key Laboratory of Solid Lubrication, Lanzhou Institute of Chemical Physics, Chinese Academy of Sciences, Lanzhou 730000, P.R. China

E-mail address: xbyan@licp.cas.cn

S. F. Zhang, Prof. A. M. Ren

State Key Laboratory of theoretical and computational chemistry, Jilin University, Jilin 130023, P. R. China

M. He, Prof. S. Xu

State Key Laboratory for Oxo Synthesis and Selective Oxidation, Lanzhou Institute of Chemical Physics, Chinese Academy of Sciences, Lanzhou 730000, P. R. China

E-mail address: xushan@licp.cas.cn

Dr. P. Zhang, M. He

University of Chinese Academy of Sciences, Beijing, 100039, P.R. China

1. Experimental Section

1.1 Materials Manufacturers

$KMnO_4$ and $Co(NO_3)_2{\cdot}6H_2O$ were purchased from Sinopharm Chemical Reagent Co., Ltd., China. Concentrated HCl and ethyl alcohol were produced by Tianjin Chemical Reagent Co., Ltd., China. Carbon paper (Toray, TDP-H060) was purchased from Shanghai Hesen Electric Co., Ltd., China. The used electrolyte (1M $LiCF_3SO_3$ in TEGDME) was purchased from Beijing Institute of Chemical Reagents and its water content was about 14 ppm.

1.2 Materials Preparation

Preparation of CP-MnO_2: MnO_2 nanorods array was synthesized via a hydrothermal method. Typically, $KMnO_4$ (307 mg, 1.93 mmol) was dissolved in deionized water (35 mL) under magnetic stirring. Then, concentrated HCl (~650 μL) was added and stirred for 10 min to form a precursor solution that was subsequently transferred into a Teflon-lined autoclave. Before sealing, a piece of CP was fixed in the autoclave vertically. After the autoclave was treated at 140 °C for 12 h and cooled down to room temperature naturally, the resulting CP-MnO_2 was rinsed several times by deionized water and then annealed at 300 °C for 2 h in air.

Preparation of CP-Co_3O_4: Co_3O_4 nanosheets were electrodeposited on a CP substrate in a $Co(NO_3)_2{\cdot}6H_2O$ solution (0.1 M) dissolved in ethanol and deionized water with equal volumes at –0.6 V. After that, the sample was heat-treated at 300 °C for 2 h in air. The Coulomb for electrodeposition was controlled at 1 C cm^{-2}.

Preparation of CP-MnO_2-Co_3O_4: The experimental process was the same as the preparation of the single MnO_2 and Co_3O_4, just changing the pure CP substrate to the CP-MnO_2 during the electrodeposition of Co_3O_4 nanosheets, and the Coulomb of Co_3O_4 was controlled at 1 C cm^{-2} or 2.5 C cm^{-2}.

1.3 Mass calculation of Co_3O_4 through coulomb's law

The mass of Co_3O_4 was calculated by the following equation:

$$NO_3^- + 7H_2O + 8e^- \rightarrow NH_4^+ + 10OH^- \quad (1)$$

$$m = \frac{5QM}{8N_A e} \quad (2)$$

$$6Co(OH)_2 + O_2 \rightarrow 2Co_3O_4 + 6H_2O \quad (3)$$

Where Q is quantity of electricity, M is the relative molecular mass of $Co(OH)_2$, N_A is the Avogadro's constant, and e is the elementary charge.

1.4 Structural characterization

The morphology of pristine, discharged and charged electrode materials were investigated by a field emission scanning electron microscope (FESEM, JSM 6701F, JEOL, Japan). A transmission electron microscope (TEM, JEOL 2100 FEG) was further applied to characterize the microstructure of CP-MnO_2, CP-Co_3O_4 and CP-MnO_2-Co_3O_4. X-ray diffraction (XRD, Rigaku D/Max-2400, Japan) patterns of the three samples were conducted on a powder XRD system using Cu Ka radiation. FTIR spectroscopy was analyzed by an IFS120HR system, and the discharge products were pressed with KBr into pellets in a glove box filled with Ar before testing. The chemical species of CP-MnO_2-Co_3O_4 were performed on a X-ray photoelectron spectroscope (XPS, Physical Electronics, PerkinElmer PHI-5702) with 1486.6 eV radiation as the excitation source and the spectra were calibrated by using Au $4f_{7/2}$ at 84.0 eV. Nitrogen adsorption-desorption isotherms were used to investigate the porous structure of the samples using a Micromeritics ASAP 2020 volumetric adsorption analyzer at 77 K. The specific surface area was caclulated based on the Brunauer-Emmett-Teller method. Pore size distribution was determined from the adsorption isotherm based on the density functional theory (DFT). The mass loading of metal oxides on CP substrate was measured by an Inductively Coupled Plasma-Atomic Emission Spectrometry (ICP-AES, optima 2100 DV).

1.5 Electrochemical measurements

All Li-O_2 batteries were tested in a typical coin cell system with holes (1 mm diameter) on the positive side, and they were assembled in an Ar-filled glovebox using a Li foil anode, a celgard 2400 separator, an oxygen cathode, and an electrolyte with 1 M $LiCF_3SO_3$ in tetraethylene glycol dimethyl ether (TEGDME). The galvanostatic tests were operated with a voltage window of 2.0–4.5 V (vs. Li/Li^+) at ambient temperature. All experiments were performed in high-purity O_2.

2. Computational section

2.1 Computational methods

DFT calculations were carried out to understand the adsorptive mechanisms of the LiO_2 on α-MnO_2, Co_3O_4 and Li_2O_2 surfaces. The system energy calculations were performed by using the Vienna *ab initio* simulation package (VASP). The projector-augmented waves (PAW) method was used to describe the electron-ion interactions, and the spin-polarized generalized gradient approximation (GGA) with Perdew-Wang (PW91) functional was used to treat the exchange-correlation energy of the electrons. The kinetic energy cutoff on the wave function expansion was set as 450 eV. The 5×5×1 *k*-point mesh was performed using the Monkhorst-Pack scheme. Ionic relaxation was executed with the conjugated gradient method. Gaussian smearing was used with a smearing parameter of 0.2 eV for these calculations. Throughout the calculations, the dipole correction was applied to compensate for the dipole interactions. The adsorption energy ($\Delta E_{adsorption}$) of LiO_2 on the surfaces was defined as:

$$\Delta E_{adsorption} = E_{total} - E_{surface} - E_{LiO_2}$$

Here, the E_{total} is the total energy for the system; $E_{surface}$ is the energy of the clean surface and E_{LiO_2} is the LiO_2 energy in vacuum, respectively.

2.2 Description of the calculation detail

Previous experimental results suggested that the facet perpendicular to the central axis of the Li_2O_2 is the (0001) surface, and the oxygen-rich (0001) surface was verified as the most stable surface in the Wulff construction of Li_2O_2 in previous DFT calculations. Thus, the oxygen-rich (0001) surface was chosen to represent the surface of Li_2O_2 crystals. The adsorption energy of LiO_2 on the (0001) surface of Li_2O_2 is calculated about –1.27 eV, which is similar to the solvation energy of LiO_2 in TEGDME (Figure 3a and 3b). In the stable adsorption configuration, the two oxygen anions of LiO_2 adsorb on the neighboring two Li^+ of Li_2O_2 and the Li^+ in LiO_2 adsorbs on the neighboring oxygen site of Li_2O_2, respectively.

We constructed slab models of the MnO_2 surface with the (020) and (110) orientations. MnO_2 was composed by the MnO_6 octahedron which formed the 2×2 frame in α-MnO_2. For the (110) plane, the stable result is –3.64 eV, and the adsorption geometry is shown in Figure 3c. In this configuration, LiO_2 molecule lies almost perpendicular to the MnO_2 (110) surface terminated with O atoms. The Li-end of the molecule is readily linked with three neighboring lattice O^{2-} ions. For the (020) plane, the stable configuration has the adsorption energy -3.82 eV and is schematically shown in Figure 3d. In this case, LiO_2 forms three connections with the MnO_2 (020) surface, corresponding to the oxygen end of LiO_2 located on top of Mn^{4+} sites and Li^+ formed bonds with two equivalent O^{2-} sites.

We constructed slab models of the Co_3O_4 surface with the (110), (112), (111) and (311) orientations. Co_3O_4 has two Co^{3+} cations at the octahedral sites and two twofold and two threefold coordinated O^{2-} anions (O2c and O3c) in surface slab model. The adsorption of molecular LiO_2 follows some general patterns, in which LiO_2 oxygen atoms interact with surface Co cations and the Li atom interacts with surface basic O^{2-} sites. However, different interaction modes have been observed for each Co_3O_4.

Co_3O_4 O-(111): The most stable structure has adsorption energy of –3.10 eV and is displayed in Figure 3e. In the resultant geometry, the LiO_2 molecule is attached to surface oxygen sites through one oxygen end and Li cation. The Li cation and the oxygen ion adsorb on the same oxygen anion of Co_3O_4.

Co_3O_4 (112): The configuration with stable adsorption energy (–3.77 eV) schematically shown in Figure 3f, has the two oxygen end adsorbing on the neighboring two Co^{3+} sites and Li^+ adsorbing on neighboring oxygen site.

Co_3O_4 (110): The DFT-calculated most stable adsorption geometry for LiO_2 on Co_3O_4 (110) are shown in Figure 3g. In this case, two oxygen ends of LiO_2 located on top of Co^{3+} sites and the corresponding adsorption energy is –3.88 eV.

Co_3O_4 (311): The stable adsorption configuration of LiO_2 on Co_3O_4 (311) is shown in Figure 3h with the corresponding energy of –4.63 eV. In detail, the oxygen ending of LiO_2 molecule adsorbing on the Co^{3+} site and the Li^+ adsorbing on the oxygen site that contacts to three Co ions.

3. Tables

Table S1 The loading mass of electrode materials on carbon paper (CP), specific surface area (SSA) and correspondences between applied current density based on SSA (1.40 mA m^{-2}) and the mass of loading metal oxides.

	CP	CP-MnO_2	CP-Co_3O_4	CP-MnO_2-Co_3O_4
Mass loading (ICP measurement, mg cm^{-2})	–	0.85	0.51	1.36 (0.85 + 0.51)
BET specific surface are (SSA, m^2 g^{-1})	2.5	21.31	74.73	71.18
Current based on BET SSA (1.40 mA m^{-2})		30 mA g^{-1}	104 mA g^{-1}	100 mA g^{-1}

Table S2 Cpacity of CP-MnO_2, CP-Co_3O_4 and CP-MnO_2-Co_3O_4 electrodes at different currents normalizd by electrode area and that normalizd by metal oxides mass.

		Current 1		Current 2		Current 3	
		results based on mass (mA g^{-1}; mAh g^{-1})	results based on area (μA cm^{-2}; mAh cm^{-2})	results based on mass (mA g^{-1}; mAh g^{-1})	results based on area (μA cm^{-2}; mAh cm^{-2})	results based on mass (mA g^{-1}; mAh g^{-1})	results based on area (μA cm^{-2}; mAh cm^{-2})
CP-MnO_2 (0.85 mg cm^{-2})	Current	52	44.20	104	88.40	311	264.35
	Capacity	2195	1.87	1543	1.31	648	0.55
CP-Co_3O_4 (0.51 mg cm^{-2})	Current	51	26.01	102	52.02	306	156.06
	Capacity	2080	1.06	1445	0.74	551	0.28
CP-MnO_2-Co_3O_4 (1.36 mg cm^{-2})	Current	51	69.36	103	140.08	309	420.24
	Capacity	5940	8.08	4850	6.60	3543	4.82
	Current	618	840.48	1236	1680.96	1647	2239.92
	Capacity	3451	4.69	2592	3.53	1588	2.16

4. Figures

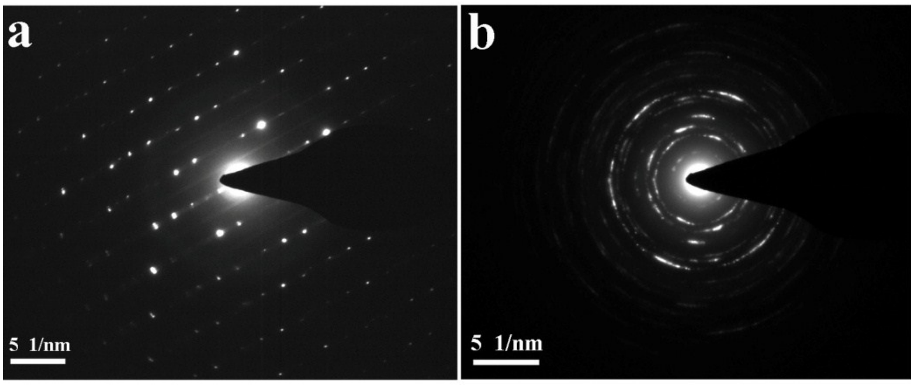

Figure S1 (a) SEAD patterns of the α-MnO_2 nanorod in Figure 1c and (b) the Co_3O_4 nanosheet in Figure 2c.

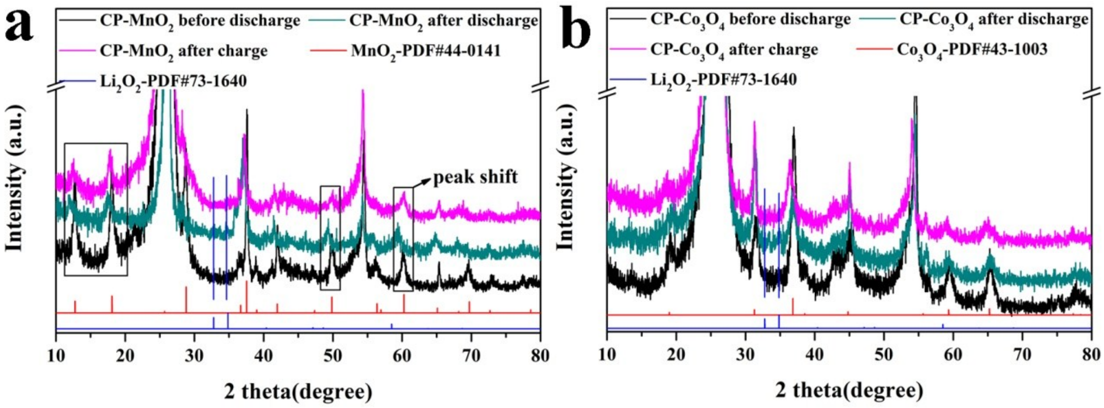

Figure S2 XRD patterns of as prepared Cp-MnO_2, discharged CP-MnO_2 and charged CP-MnO_2 electrodes (a), and XRD patterns of as prepared Cp-Co_3O_4, discharged CP-Co_3O_4 and charged CP-Co_3O_4 electrodes (b).

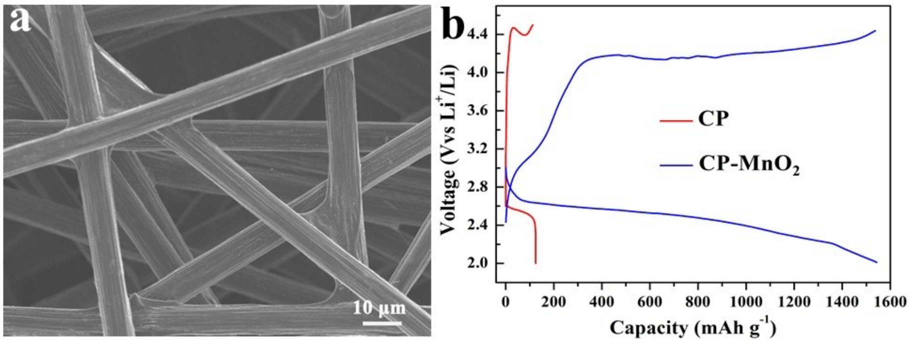

Figure S3 (a) SEM image of CP substrate. (b) Charge–discharge profiles of CP and CP-MnO_2 at the current density of 4.8 mA $m^{-2}{}_{SSA}$ (same as 104 mA g^{-1} for CP-MnO_2 and 12 mA g^{-1} for CP). It should be noted that, to confirm the critical role of loading active material, the capacity was normalized to the mass of MnO_2 in CP-MnO_2 for both of the two electrodes.

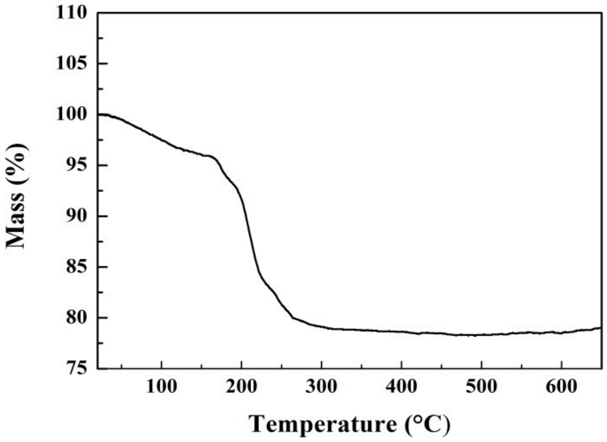

Figure S4 TGA curve of $Co(OH)_2$ under air atmosphere.

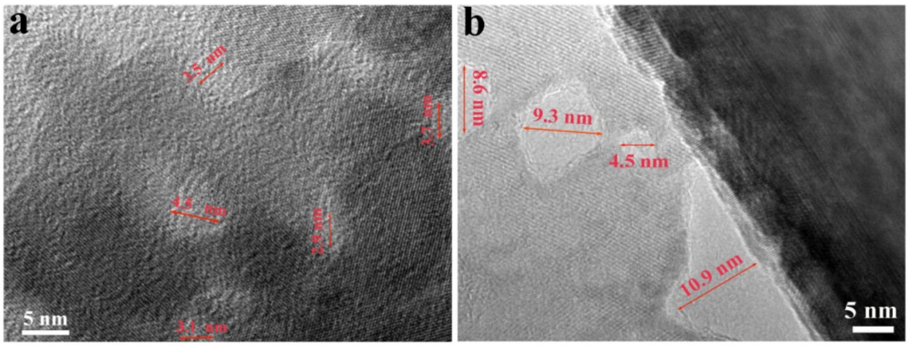

Figure S5 HRTEM images of CP-Co_3O_4 (a) and CP-MnO_2-Co_3O_4 (b).

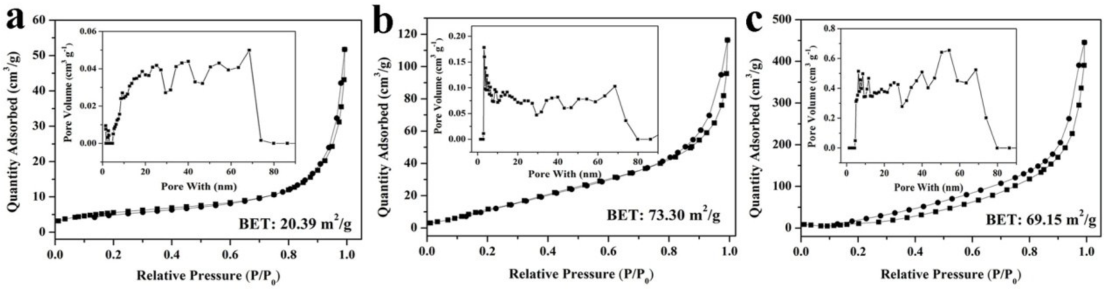

Figure S6 Nitrogen adsorption–desorption isotherms and the pore size distributions (inset) of CP-MnO_2 (a), CP-Co_3O_4 (b) and CP-MnO_2-Co_3O_4 (c) samples.

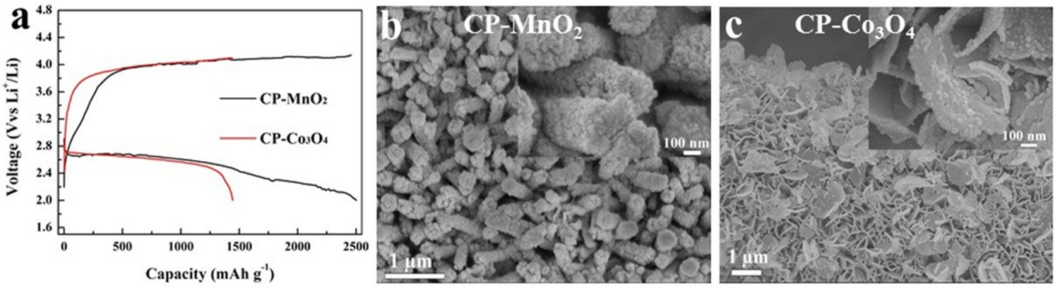

Figure S7 (a) Charge-discharge curves of the CP-MnO_2 (30 mA g^{-1}) and CP-Co_3O_4 (100 mA g^{-1}) electrodes at about 1.40 mA m^{-2} (based on specific surface area). (b) The morphology of discharged CP-MnO_2 (b) and CP-Co_3O_4 (c) electrodes at about 1.40 mA m^{-2}.

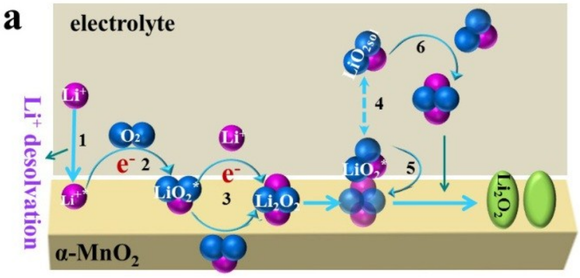

$$Li^{+}_{sol} \rightarrow Li^{+} * \quad (1)$$

$$Li^{+} * + O_2 + e^{-} \rightarrow LiO_2^{*} \quad (2)$$

$$LiO_2^{*} + LiO_2^{*} \rightarrow Li_2O_2 + O_2;\ LiO_2^{*} + Li^{+}_{sol} + e^{-} \rightarrow Li_2O_2(\text{sur - nucleation}) \quad (3)$$

$$\text{On } Li_2O_2 \text{ surfaces}:\ LiO_{2sol} \leftrightarrow LiO_2^{*} \quad (4)$$

$$LiO_2^{*} + LiO_2^{*} \rightarrow Li_2O_2 + O_2;\ LiO_2^{*} + Li^{+} + e^{-} \rightarrow Li_2O_2(\text{on sur - seeds}) \quad (5)$$

$$LiO_{2sol} \rightarrow (nLiO_2)_{sol} \rightarrow \frac{n}{2}Li_2O_2 + \frac{n}{2}O_2(\text{on sur} - \text{seeds}) \quad (6)$$

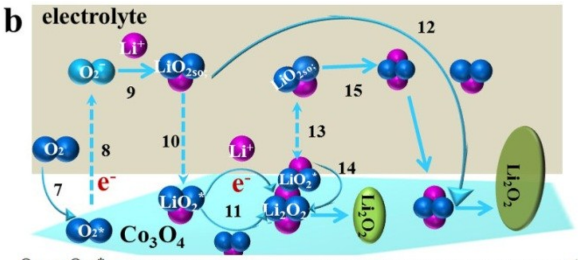

$$O_2 \rightarrow O_2 * \quad (7)$$

$$O_2 * + e^{-} \rightarrow O^{-}_{2sol} \quad (8)$$

$$O^{-}_{2sol} + Li^{+}_{sol} \rightarrow LiO_{2sol} \quad (9)$$

$$\text{On } Co_3O_4 \text{ surfaces}:\ O^{-}_{2sol} + Li^{+}_{sol} \rightarrow LiO_2^{*} \quad (10)$$

$$LiO_2^{*} + LiO_2^{*} \rightarrow Li_2O_2 + O_2;\ LiO_2^{*} + Li^{+} + e^{-} \rightarrow Li_2O_2(\text{sur} - \text{seeds}) \quad (11)$$

$$\text{In electrolyte}: LiO_{2sol} \rightarrow (nLiO_2)_{sol} \rightarrow \frac{n}{2}Li_2O_2 + \frac{n}{2}O_2(\text{sol} - \text{seeds}) \quad (12)$$

$$\text{On } Li_2O_2 \text{ surfaces}: LiO_{2sol} \leftrightarrow LiO_2 * \quad (13)$$

$$LiO_2^{*} + LiO_2^{*} \rightarrow Li_2O_2 + O_2;\ LiO_2^{*} + Li^{+} + e^{-} \rightarrow Li_2O_2(\text{on sur} - \text{seeds}) \quad (14)$$

$$LiO_{2sol} \rightarrow (nLiO_2)_{sol} \rightarrow \frac{n}{2}Li_2O_2 + \frac{n}{2}O_2(\text{on sol} - \text{seeds}) \quad (15)$$

Figure S8 Schematic illustrations of the discharging mechanism for α-MnO_2 nanorod (a) and Co_3O_4 nanosheet (b). The equations are the corresponding reaction process of MnO_2 (1-6) and Co_3O_4 (7-15). The characters of "sol-nucleation/seeds" and "sur-seeds" represent the seeds formed through solution and surface, respectively.

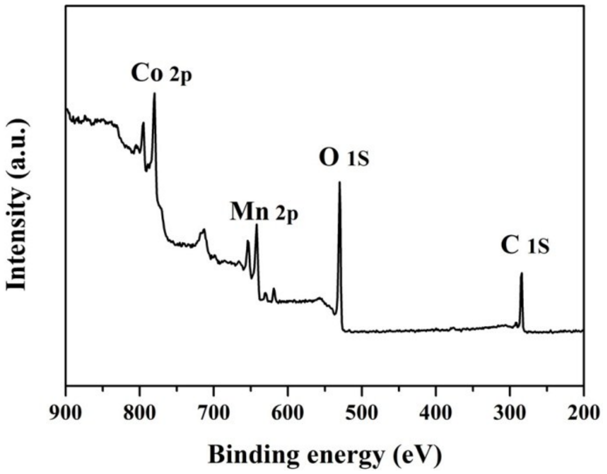

Figure S9 XPS spectrum of CP-MnO_2-Co_3O_4.

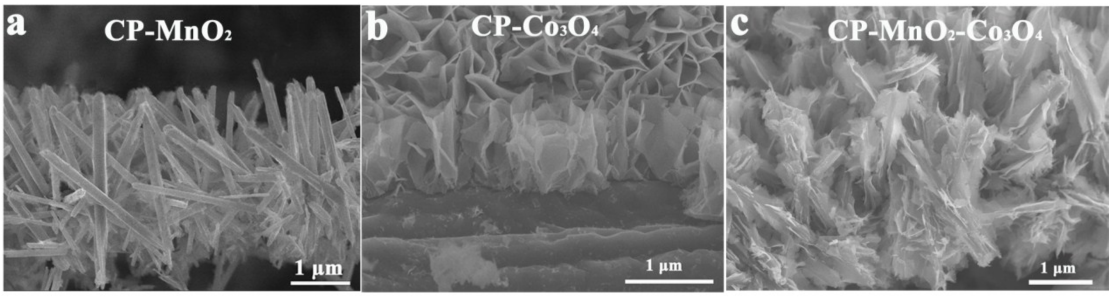

Figure S10 SEM image of CP-MnO_2, CP-Co_3O_4 and CP-MnO_2-Co_3O_4 sample from side view.

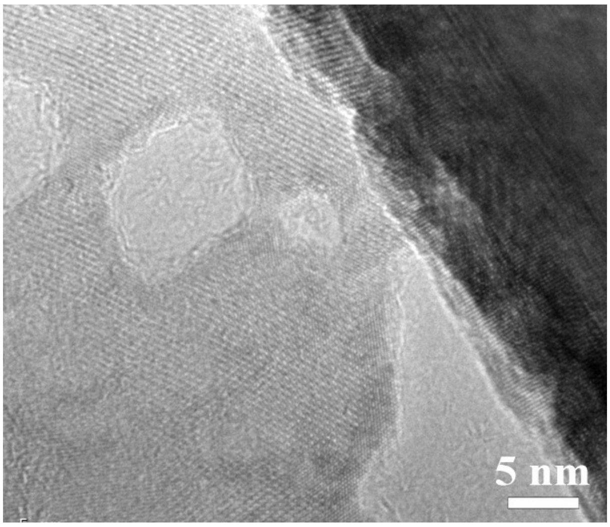

Figure S11 TEM image of the interface between MnO_2 nanorod and Co_3O_4 nanosheet.

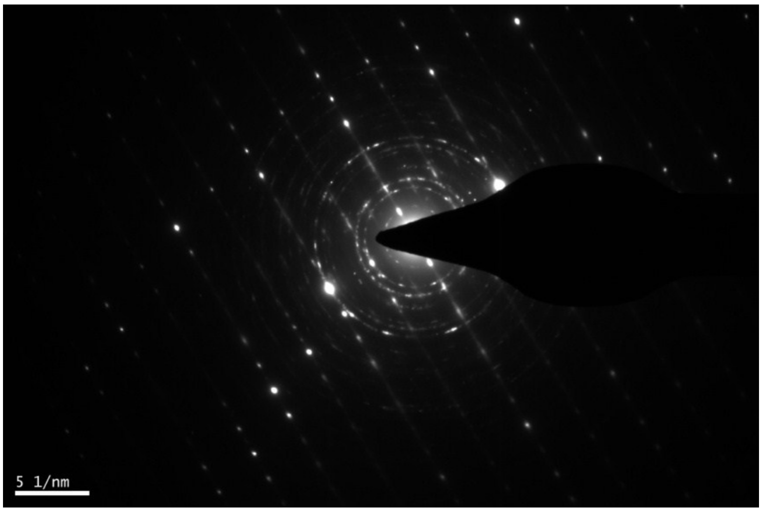

Figure S12 SEAD pattern of CP-MnO_2-Co_3O_4 sample.

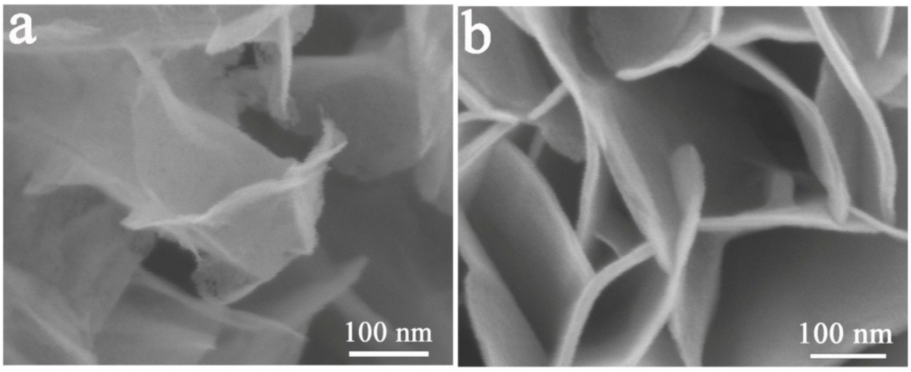

Figure S13 SEM images of CP-MnO_2-Co_3O_4 electrode (a) and CP-Co_3O_4 electrode (b) at a high magnification.

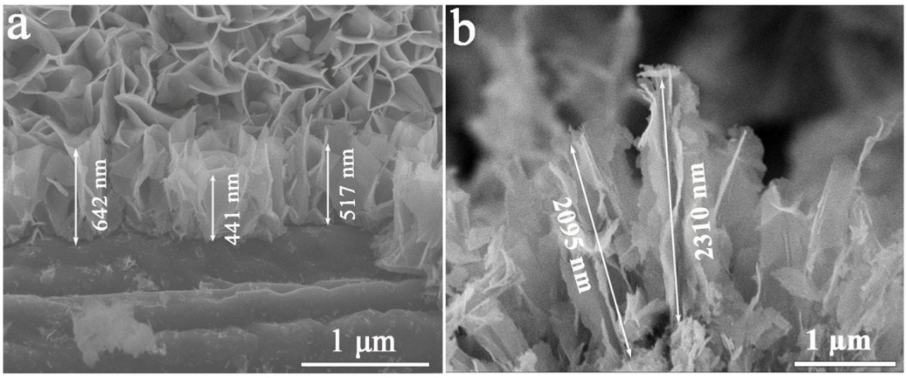

Figure S14 The side view SEM images of CP-Co_3O_4 (a) and CP-MnO_2-Co_3O_4 (b) samples, indicating the much increased height of Co_3O_4 in the composite sample.

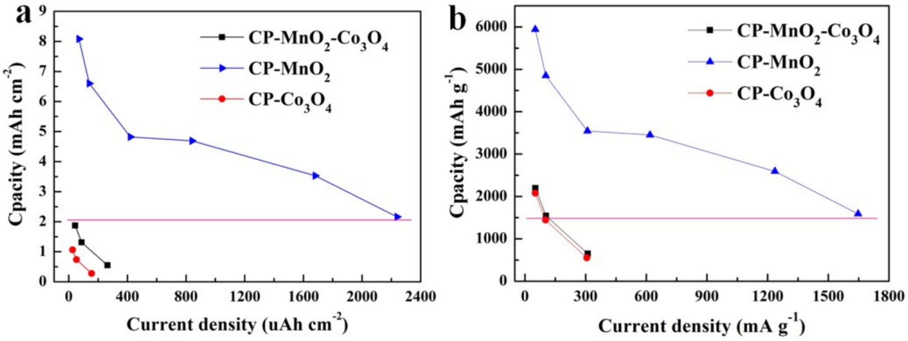

Figure S15 The comparison of electrochemical performance of CP-Co_3O_4, CP-MnO_2 and CP-MnO_2-Co_3O_4 samples normolized by electrode area (a) and metal oxides mass (b).

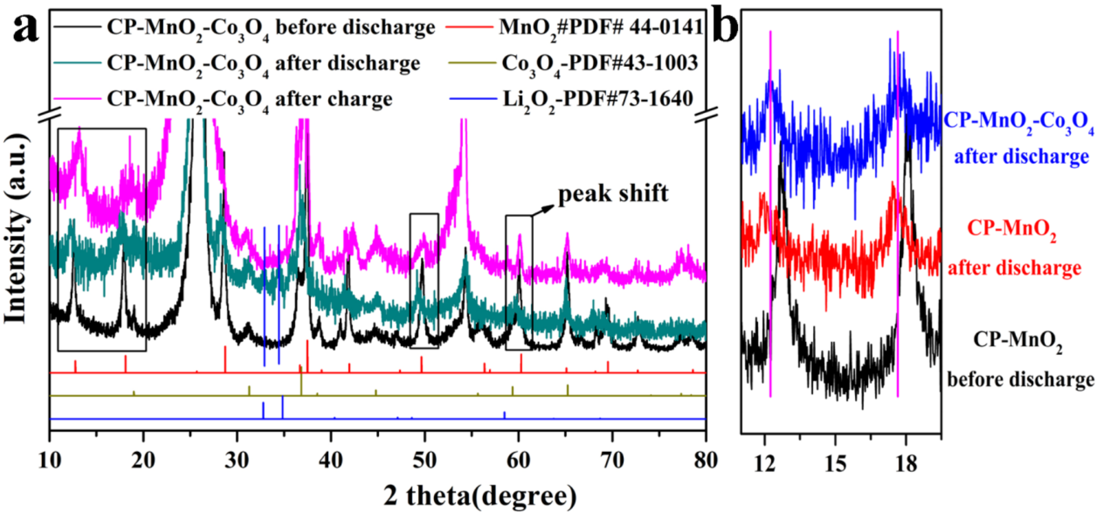

Figure S16 (a) XRD patterns of CP-MnO_2-Co_3O_4 electrode at before discharge, after full discharge and after recharge state. (b) The comparison of peak shifts of CP-MnO_2 and CP-MnO_2-Co_3O_4 electrodes after discharge.

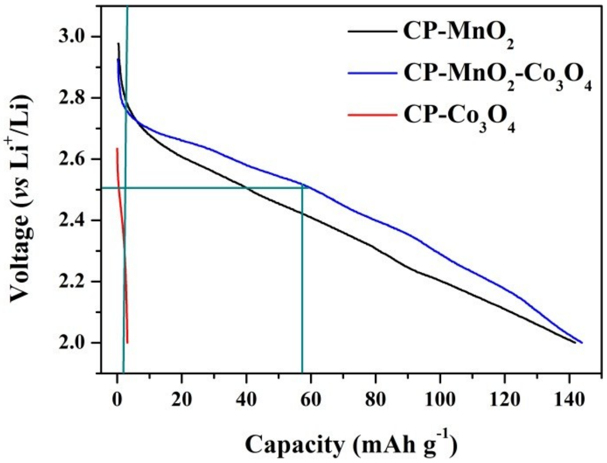

Figure S17 Discharging profiles of the three electrodes tested in Ar atmosphere. It should be noted that the specific capacity of CP-MnO_2-Co_3O_4 electrode was calculated based on the MnO_2 mass.

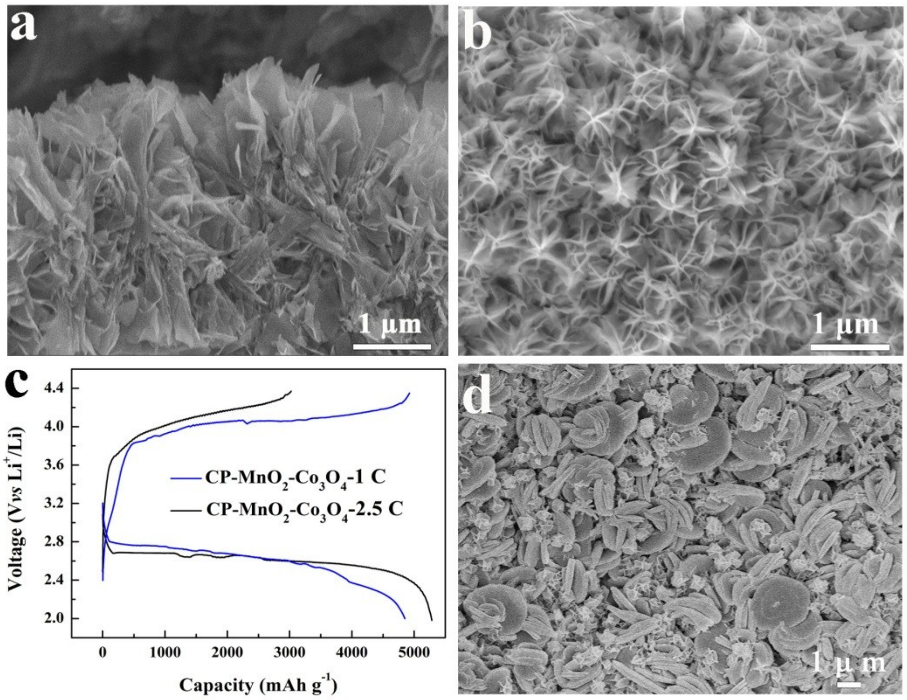

Figure S18 SEM image of CP-MnO_2-Co_3O_4-2.5 C electrode from side view (a) and top view (d).(c) Charge–discharge curves of the composite electrodes with different Co_3O_4 loading at ~103 mA g^{-1}. (d) SEM image of discharged CP-MnO_2-Co_3O_4-2.5 C electrode. The loading mass of MnO_2 and Co_3O_4 in CP-MnO_2-Co_3O_4-2.5 C sample is 0.85 mg cm^{-2} and 1.27 mg cm^{-2}, respectively.

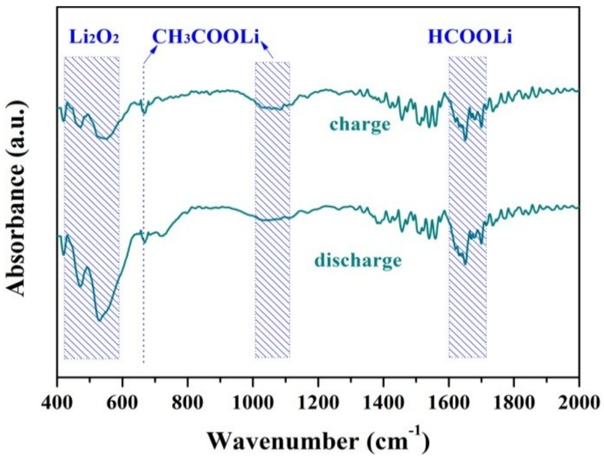

Figure S19 FTIR spectra of CP-MnO_2-Co_3O_4 electrode at 30th cycle under the limited capacity of 1030 mAh g^{-1}.

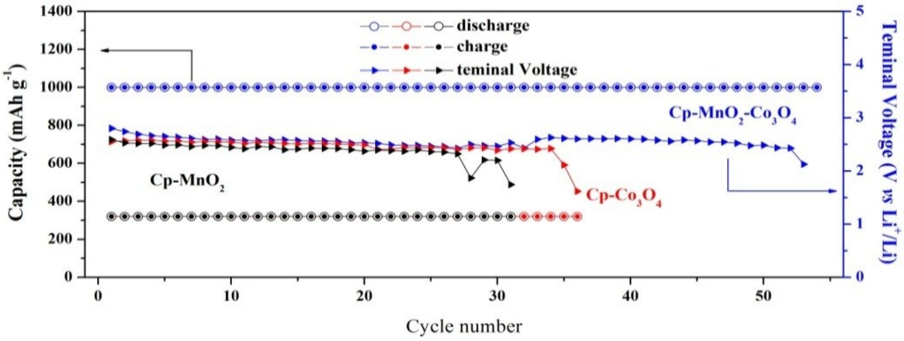

Figure S20 The cyclic stability of the three electrodes under the limited capacity corresponding to ~21% of their full capacity at ~103 mA g^{-1}.

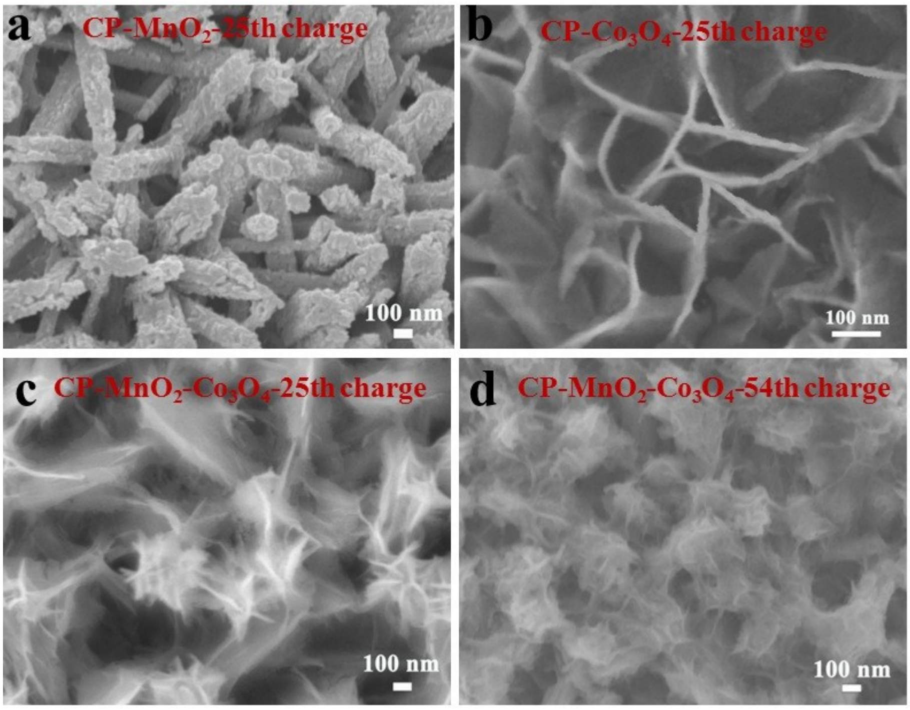

Figure S21 SEM images of charged CP-MnO_2 (a) and CP-Co_3O_4 (b) at 25th cycle, SEM images of charged CP-MnO_2-Co_3O_4 electrode at the 25th (c) and 54th (d) cycle.

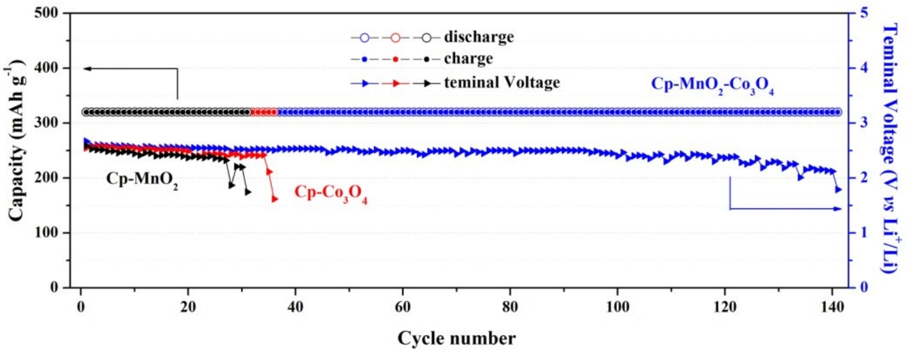

Figure S22 The cyclic stability of the three electrodes with the limited capacity of 320 mAh g^{-1} at ~103 mA g^{-1}.

Though Li^+ can intercalate into the MnO_2 crystal structure, the capacity provided by lithiation is only about 36 mAh g^{-1} above 2.5 V based on the total mass of MnO_2 and Co_3O_4 (Figure S17). When CP-MnO_2-Co_3O_4 electrode is tested in oxygen, the capacity provided by lithiation would be less than that tested in Ar atmosphere due to some MnO_6 octahedron channels being occupied by Li_xO_y. Thus, the cycle test of CP-MnO_2-Co_3O_4 electrode at 320 mAh g^{-1} can also verify its superior cycle stability.
